# Temporary anchorage device usage: a survey among Swiss orthodontists

**DOI:** 10.1186/s40510-014-0029-x

**Published:** 2014-04-01

**Authors:** Goran Markic, Christos Katsaros, Nikolaos Pandis, Theodore Eliades

**Affiliations:** 1 Clinic for Orthodontics and Paediatric Dentistry, Centre of Dental Medicine, University of Zurich, Plattenstrasse 11, Zurich 8032, Switzerland; 2 Department of Orthodontics and Dentofacial Orthopaedics, University of Bern, Freiburgstrasse 7, Bern 3010, Switzerland

**Keywords:** Temporary anchorage device, TAD, Mini-screw, Mini-implant, Palatal implant, Mini-plate, Infrazygomatic arch, Position, Treatment planning, Survey

## Abstract

**Background:**

The aim of the survey was to obtain information on the treatment plan preferences, mechanics and characteristics of temporary anchorage device (TAD) application using a single case presented to orthodontists in Switzerland.

**Methods:**

A structured questionnaire to be completed by all study participants with case-specific (treatment plan including mechanics and TAD usage) and general questions (general fixed appliance and TAD usage as well as professional, educational and demographic questions) together with an orthodontic borderline case was utilised. The case was a female adult with dental Class II/2, deep bite and maxillary anterior crowing, who had been treated in childhood with extraction of four premolars and fixed appliance followed by wisdom tooth extraction.

**Results:**

The response rate was 24.4% (108 out of 443). The majority (96.3%, 104) proposed comprehensive treatment, while 3.7% (4) planned only alignment of maxillary teeth. 8.3% (9) included a surgical approach in their treatment plan. An additional 0.9% (1) combined the surgical approach with Class II mechanics. 75.1% (81) decided on distalization on the maxilla using TADs, 7.4% (8) planned various types of Class II appliances and 3.7% (4) combined distalization using TADs or headgear with Class II appliances and surgery. Palatal implants were the most popular choice (70.6%, 60), followed by mini-screws (22.4%, 19) and mini-plates on the infrazygomatic crests (7.0%, 6). The preferred site of TAD insertion showed more variation in sagittal than in transversal dimension, and the median size of mini-screws used was 10.0-mm long (interquartile range (IQR) 2.3 mm) and 2.0-mm wide (IQR 0.3 mm).

**Conclusions:**

Distalization against palatal implants and then distalization against mini-screws were the most popular treatment plans. Preferred site for TAD insertion varied depending on type and size but varied more widely in the sagittal than in the transversal dimension.

## Background

Temporary anchorage devices (TADs) have become an established treatment modality in orthodontics and have facilitated successful treatment of more complex orthodontic cases [1] such as borderline adult Class II and asymmetric cases.

In growing Class II cases, growth modification or extraction is often the therapy of choice, whereas in adults, orthognathic surgery and orthodontic camouflage treatment, including Herbst appliance treatment [2-4], remain the only treatment options. Several factors can be identified influencing the choice of therapy for an adult Class II case: severity of skeletal and dental discrepancy, amount of crowding especially in the lower jaw, periodontal condition, expected stability, age and the willingness of the patient to undergo orthognathic surgery.

In borderline cases without severe skeletal discrepancies, orthodontic camouflage treatment may be an acceptable choice compared to orthognathic surgery [5,6]. The following scenarios in orthodontic camouflage therapy can be considered: extractions and active distalization in the upper jaw, extractions in both jaws, intermaxillary Class II mechanics, bite-jumping appliances, such as Herbst appliance, and a combination of these techniques.

Before TADs became available, distalization in the upper jaw had to rely on extra-oral traction using headgear and in which patient compliance was detrimental to the success of the therapy. With the introduction of TADs, patient cooperation became less important with the added benefit of almost absolute anchorage [7].

The use of TADs has seen a dramatic increase, and two recent surveys among orthodontists in the USA revealed that over 70% to 91% are using some form of TADs in their practices or during their residency programs [8,9]. Although TADs are presently a viable treatment option, to the best of our knowledge, no information about the use of TADs among orthodontists in Switzerland is available.

The main objective of the study was to assess the distribution of treatment plans concerning anchorage, extractions and orthognathic surgery as well as the associated mechanics among orthodontists in Switzerland to solve this borderline case. In case of skeletal temporal anchorage device usage, the secondary aim was also to assess their types and positions. The third aim was to assess general professional, educational and demographic information as well as information about TAD usage from orthodontists in Switzerland and to test the hypothesis if there were any associations between general baseline characteristics of survey participants, the chosen therapy and TAD usage for the presented borderline case.

## Methods

**Figure 1:**
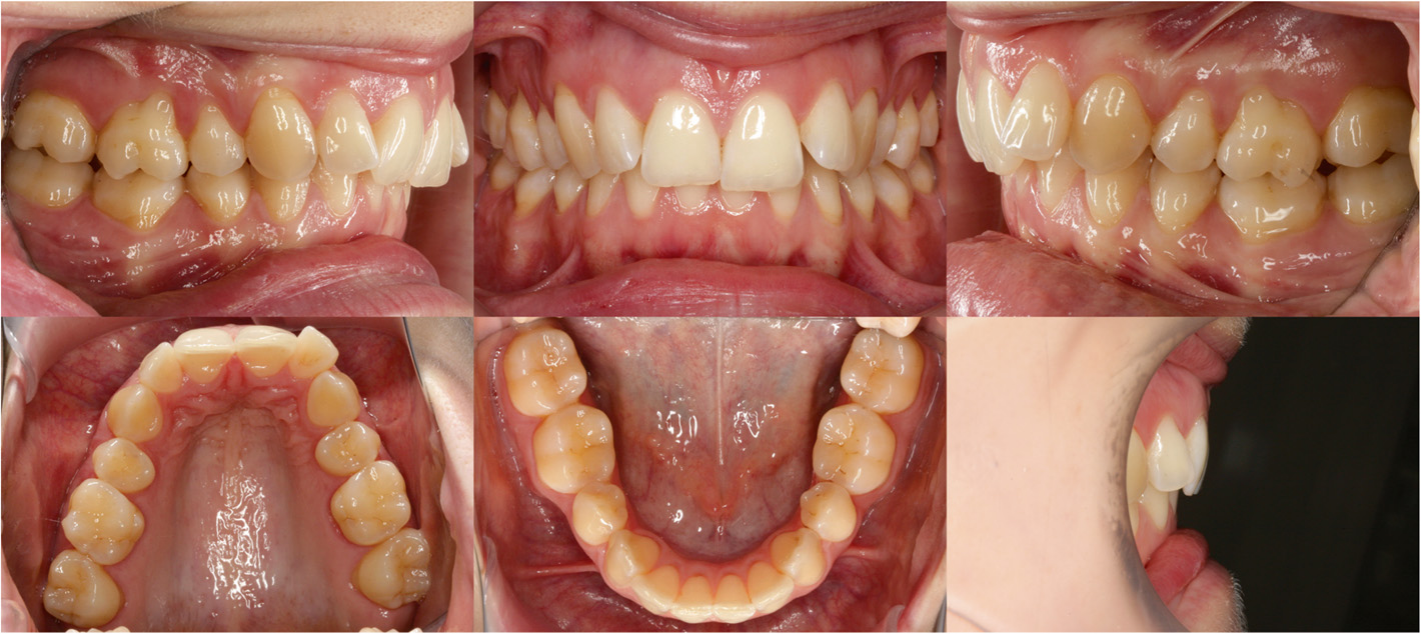
Pretreatment intra-oral photographs.

**Figure 2:**
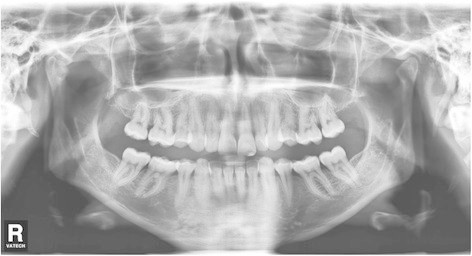
Pretreatment OPG.

**Figure 3:**
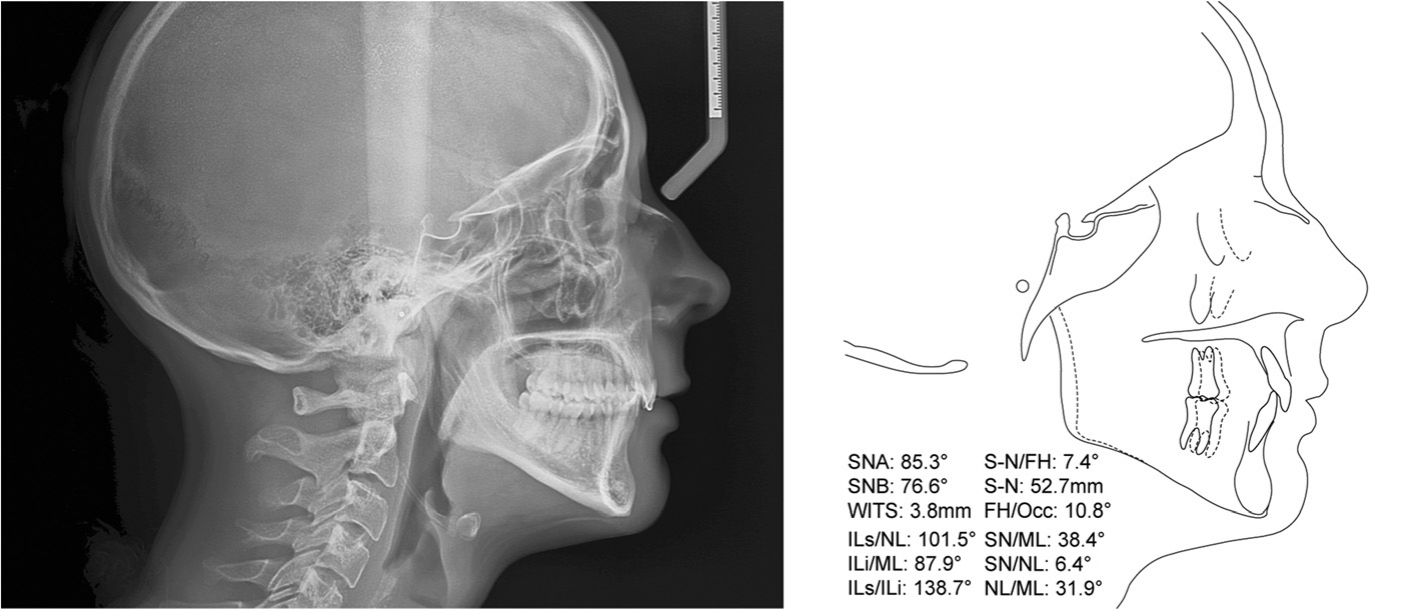
Pretreatment lateral cephalogram and its tracing with the most common measurements.

The survey was based on an internet webpage, where the case of a young woman was presented at the website of the Department of Orthodontics of the University of Zürich. The pretreatment records provided were oral photographs (Figure 1), orthopantomogram (OPG) (Figure 2) and lateral cephalogram with a tracing including most common skeletal and dental measurements (Figure 3). The webpage allowed an enlarged view of all records for a detailed identification of the anatomy. The patient agreed to participate in the study and consented to the open access of the webpage.

The case showed a young, healthy female 29 years of age. Her chief complaints were the irregularities of her teeth and the bite situation. The patient had already had treatment in childhood with extraction of four premolars and fixed appliances followed by wisdom tooth extraction. No active periodontal or carious lesions were present, and all teeth were vital. The oral photographs indicated buccal gingival recessions, a Class II/2 malocclusion, deep bite and anterior crowing in the upper arch. The profile, which was not a concern to the patient, showed a moderate mandibular retrognathia and concavity.

**Figure 4:**
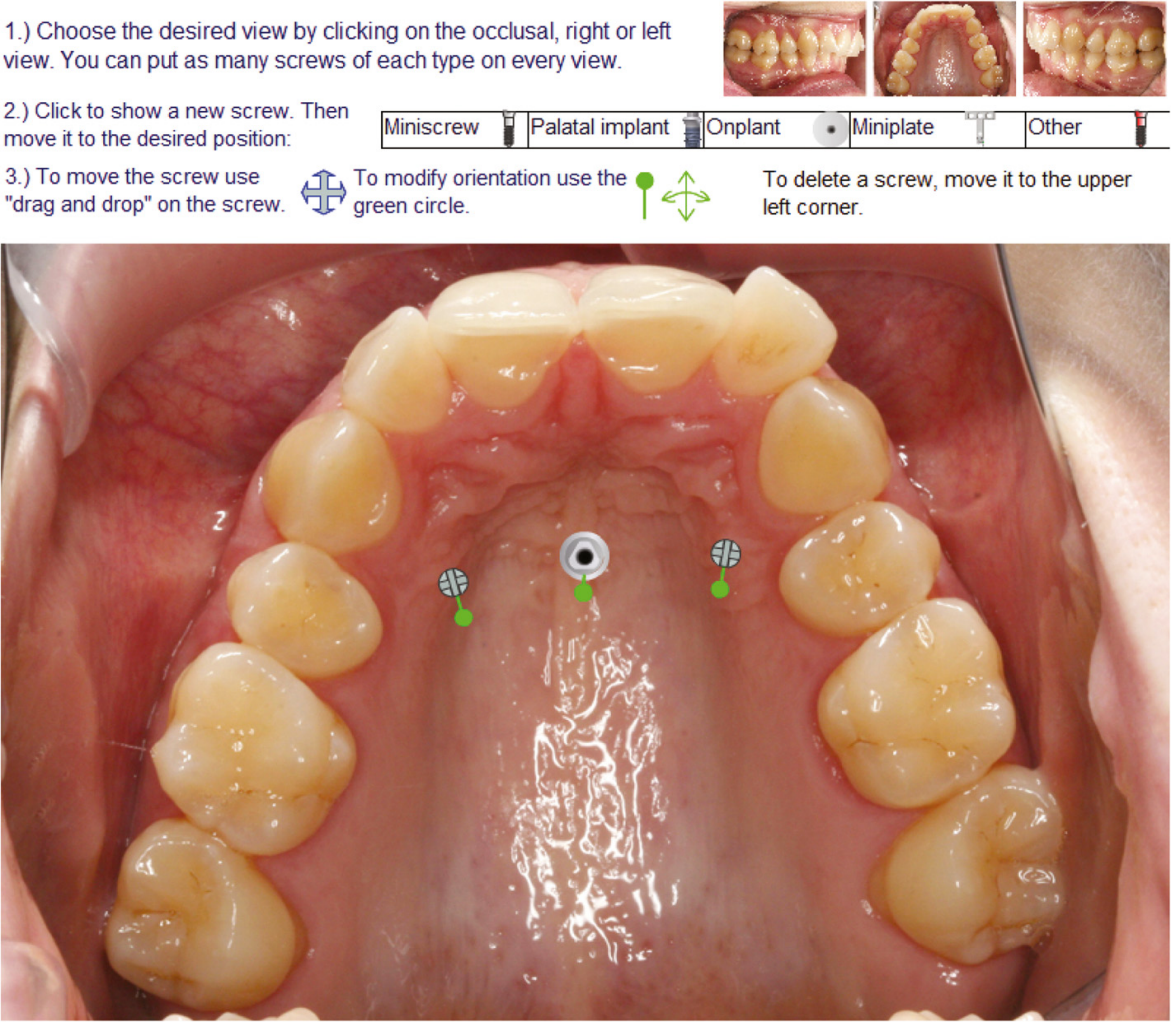
**Interactive application for TAD placement.** One example of the occlusal view of the upper jaw with one palatal implant and two mini-screws.

Together with the case documentation, a structured questionnaire (Additional file 1) with an interactive application for TAD placement (Figure 4) was available on the website. The application allowed the placement of any number of different types of TADs (mini-screws, palatal implants, onplants, mini-plates and other TADs) on each view (occlusal view of the upper arch, left buccal and right buccal view). Additionally, the application permitted the orientation of each TAD to be adjusted in mesio-distal, bucco-oral and rotational dimensions to conform to clinical usage.

All members of the Swiss Orthodontic Society and orthodontists and postgraduates working at the universities in Switzerland were invited to participate in this survey. The Swiss Orthodontic Society approved the use of their address database for the current study, and each member received a survey participation letter. The survey letter included a randomly created unique alpha-numeric eight-character code that had to be entered on the webpage to allow filtering out double records. Although this code was not mandatory, the orthodontists were kindly asked to use it to increase the quality of the acquired data. If someone refused to enter the code, at least the date of birth and the initials had to be provided. The webpage was online, and data were collected from the beginning of May to the end of November 2012 with a reminder letter sent in September 2012.

All orthodontists were kindly asked to visit the website and to fill out the web form. All fields had to be filled out. The questionnaire was split into a first section specific to the case presented and the second section that contained general questions. All questions are presented in Additional file 1.

All free text sections were evaluated by the first author (GM) and converted to values and categories suitable for statistical analysis. Due to the extremely high level of details resulting in a huge number of similar concepts with only little difference, similar treatment concepts were combined and detailed information about the mechanics was not included in the statistical analysis. As an example, all distalization cases using mini-screws were combined, independent of the manufacturer of the mini-screws. In the case of multiple treatment options proposed, only the first treatment option was included in the statistical analysis. Unrealistic positions of TADs (e.g. palatal implant positioned in the upper lip) were ignored.

The central position of a centroid for each screw type and subgroup was assessed by means of *x* and *y* coordinates separately. The calibration between the occlusal photograph (pixel coordinates of screw positions) and the real-world coordinates (millimetre coordinates) was performed by measuring the distance between the palatal cusps of 15 and 25 on the photograph (pixel distance) and on the plaster model (millimetre distance).

### Statistical analysis

The following outcomes were considered: treatment plan choices, mechanics, number and type of skeletal anchorage devices. The distributions of treatment plans and mechanics as well as general questions were presented as counts and relative frequencies.

Associations between selected general questions shown in the header of Table 1 (predictors) and main categories of chosen treatment options and TAD types used for distalization (outcomes) were analysed by means of multinomial logistic regression. Additionally, to quantify how well the observed outcomes were replicated by the model, the Cox and Snell pseudo *R*^2^ index was provided. Normality assumptions for continuous variables were tested using Shapiro-Wilk and Kolmogorov-Smirnov tests.

**Table 1:** **Frequencies of categorical variables in general questions from all responders: 108 (100**%**)**

	**Frequency**	**Percentage**
Orthodontic technique		
Straight wire (sliding)	66	61.1
Straight wire (loop mechanics)	26	24.1
Standard edge wise	12	11.1
All other systems	4	3.7
Self-ligation		
Yes	62	57.4
No	46	42.6
Bracket slot sizes or types		
0.018″	43	39.8
0.022″	64	59.4
0.018″ (front) and 0.022″ (back)	1	0.9
Country where specialisation was obtained		
Switzerland	84	77.8
Germany	7	6.5
Other	6	5.5
Undefined	11	10.2
University where specialisation was obtained		
Zürich	43	39.8
Bern	23	21.3
Basel	8	7.4
Geneva	11	10.2
Outside of Switzerland	11	10.2
Undefined	12	11.1
Working in private practice as		
Practice owner	64	59.3
Practice partner	12	11.1
Assistant or associate	13	12.0
Not working in private practice	16	14.8
Other	3	2.8
Gender		
Male	81	75.0
Female	27	25.0

Continuous outcomes (number of skeletal anchorage devices such as palatal implants, mini-screws, mini-plates on the infrazygomatic crest and onplants, screw positions and dimensions) were presented using either the median, interquartile range (IQR), percentiles (25th and 75th), minimum and maximum values or the mean, standard deviation (SD), and minimum and maximum values. Additionally, the data were also grouped by TAD type used in the treatment plan.

All data were coded in Excel 2010 (version 14.0.6112.5000, Redmond, WA, USA) and analysed in SPSS (version 20.0.0, Chicago, IL, USA). *P* values smaller than 0.05 were considered statistically significant.

## Results and discussion

### Results

A total of 463 letters were mailed, and the percentage of undelivered letters was 4.3% (20). One recipient refused to fill out the questionnaire because of too little information and incomplete pretreatment records in his opinion, 24.4% (108/443) completed the questionnaire and 95.4% (103) used the code provided in the letter.

All proposed treatments were non-extraction and included fixed appliances. The majority of the responders (96.3%, 104) proposed a comprehensive treatment, while 3.7% (4) planned only alignment without Class II correction. Distalization in the upper jaw using TADs to correct Class II cases was chosen by 75.1% (81/108); 70.4% (57/81) used palatal implants, 22.2% (18/81) used mini-screws and 7.4% (6/81) used mini-plates on the infrazygomatic crests. A surgical approach was included in 8.3% (9/108) of the treatment plans; 77.8% (7/9) decided to use a sagittal split osteotomy, 11.1% (1/9) a LeFort I osteotomy and 11.1% (1/9) a combination of SARPE, LeFort I and sagittal split osteotomy. Class II mechanics to correct the sagittal relationship was planned in 7.4% (8/108) with 50.0% (4/8) using Herbst appliance, 37.5% (3/8) using springs and 12.5% (1/8) using elastics. The summary of all treatment options is shown in Table 2, and the descriptive statistics of the general questions section are shown in Table 1.

**Table 2:** Summary of treatment options

	**Count**	**Percentage of all treatments**	**Relative percentage**
All (non-extraction with fixed appliance)	108	100	
Distalization only	81	75.1	100
Palatal implant	57	52.8	70.4
Mini-screws	18	16.7	22.2
Mini-plates on infrazygomatic crests	6	5.6	7.4
Distalization combined with Class II mechanics	4	3.7	100
Palatal implant	2	1.9	50.0
Headgear	1	0.9	25.0
Mini-screws	1	0.9	25.0
Orthognathic surgery only	9	8.3	100
Sagittal split osteotomy	7	6.5	77.8
LeFort I osteotomy	1	0.9	11.1
Sagittal split, LeFort I osteotomy and SARPE	1	0.9	11.1
Sagittal split osteotomy combined with Class II mechanics	1	0.9	
Sagittal split osteotomy combined with distalization against palatal implant	1	0.9	
Class II mechanics	8	7.4	100
Herbst appliance	4	3.7	50.0
Springs	3	2.8	37.5
Elastics	1	0.9	12.5
Alignment only without Class II correction	4	3.7	

None of the factors of the general questions section were associated with the main treatment selections (Table 3). In the subgroup using TADs for distalization, the following general questions were associated with the TAD type: the orthodontic technique, self-ligation usage, bracket slot size, country of specialisation, university of specialisation and the number of mini-screws placed. The coefficient of determination for these associations was low showing values between 0.10 and 0.35 (Table 3).

**Table 3:** Associations between data of general questions and chosen treatment therapy with coefficient of determination

**Categories of general questions section**	**TAD type used for distalization**	**Treatment options: main categories**
Orthodontic technique (fixed appliance)	0.20*	0.21
Self-ligation	0.16*	0.07
Bracket slot sizes	0.10*	0.17
Country of specialisation	0.25*	0.10
University of specialisation	0.29*	0.27
Working in private practice as	0.20	0.15
Gender	0.05	0.08
Years working as orthodontist	0.09	0.00
Years working in private practice	0.32	0.00
Number of mini-screws placed approximately in February and March of 2012	0.35*	0.29
Number of palatal implants placed approximately in February and March of 2012	0.27	0.29
Number of mini-plates placed approximately in February and March of 2012	0.11	0.12

*P < 0.05.

In exploring the actual usage of TADs in the practices of the questionnaire responders, we found that the general usage of TADs between February and March of 2012, the survey period, was generally low (Table 4). Fifty percent of all survey participants did not use any TADs at all in this period of time (median 0.0) in their practices (75th percentile: mini-screws 3, palatal implants 1 and mini-plates to the infrazygomatic arch 0). Practitioners who recommended mini-screws for distalization in the survey case used predominantly mini-screws as skeletal anchorage between February and March of 2012 in their practices (median of 4 and 75th percentile of 12). Only one practitioner used in his practice 50 mini-screws during the period our survey. In the group of practitioners who planned palatal implants for distalization, very few TADs were used (mini-screw and palatal implants 75th percentile 2).

**Table 4:** Descriptive statistics of the amount of the placement of different TADs

	**Median**	**IQR**	**Percentiles**		**Minimum**	**Maximum**
			**25th**	**75th**		
For all participants (*n* = 108)						
Mini-screws	0.0	3.0	0.0	3.0	0	50
Palatal implants	0.0	1.0	0.0	1.0	0	10
Mini-plates	0.0	0.0	0.0	0.0	0	8
For participants who used mini-screws in their treatment concepts (*n* = 19)						
Mini-screws	4.0	12.0	0.0	12.0	0	50
Palatal implants	0.0	0.0	0.0	0.0	0	6
Mini-plates	0.0	0.0	0.0	0.0	0	4
For participants who used palatal implants in their treatment concepts (*n* = 60)						
Mini-screws	0.0	2.0	0.0	2.0	0	12
Palatal implants	1.0	2.0	0.0	2.0	0	10
Mini-plates	0.0	0.0	0.0	0.0	0	8
For participants who used mini-plates in their treatment concepts (*n* = 6)						
Mini-screws	4.5	0.0	0.0	6.0	0	6
Palatal implants	0	0.0	0.0	0.0	0	0
Mini-plates	0.0	2.0	0.0	2.0	0	2

Data from general question section of how many skeletal anchorage devices have been placed approximately in February and March of 2012 by anchorage device type. The first group shows statistics for all participants. The following three groups show statistics of subgroups based on the TAD type used in the treatment plan of the current case.

**Figure 5:**
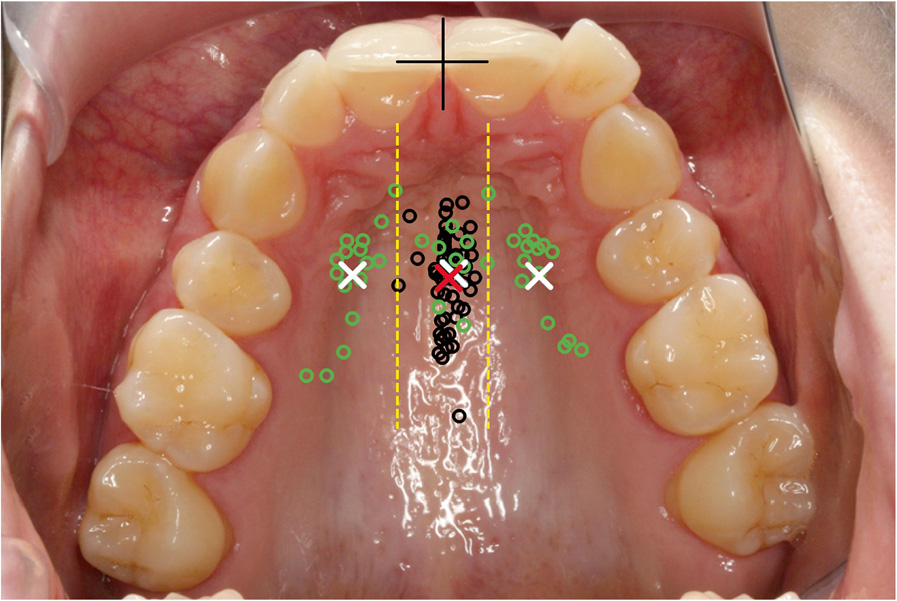
**Scattergram of distribution of palatal implants (black circles) and mini-screws (green circles).** Reference point for the measurements (black cross) defined by the incisal edge and raphe palatina mediana. Centroids of palatal implants (red cross) and left, right and centre groups of mini-screws (white crosses). Borderlines between mini-screw groups are delimited with yellow dashed lines.

The distribution of palatal implant positions (Figure 5, Table 5) showed a wide range in the sagittal plane with only little variation in the transversal dimension and with most implants positioned in the midline or slightly paramedian on the patient's left side. The positions of palatal mini-screws were divided into three subgroups: lateral left, lateral right and median. The median group had a similar distribution as the palatal implant group but with a smaller range. The lateral subgroups of mini-screws were mostly positioned along diagonally arranged lines, ventro-mesial to disto-caudal, parallel to the alveolar ridge. The ranges were similar but bigger than the values of the palatal implant group. For all groups, the *x* and *y* coordinates were normally distributed. The median length of mini-screws used was 10.0 mm with a median diameter of 2.0 mm. Mini-screw lengths and diameters were not normally distributed.

**Table 5:** Descriptive statistics of TAD positions

	**Mean ± SD**	**Median**	**Percentiles**		**Minimum**	**Maximum**
			**25th**	**75th**		
Palatal implants						
*x* coordinates (mm)	0.5 ± 1.0	0.3	0.0	1.2	−3.2	2.4
*y* coordinates (mm)	15.6 ± 3.0	15.2	14.0	17.0	10.2	25.6
Mini-screws						
Patient's right side						
*x* coordinates (mm)	−6.5 ± 1.6	−6.9	−7.4	−5.6	−9.8	−3.4
*y* coordinates (mm)	15.4 ± 3.8	14.4	12.9	17.4	9.3	22.7
Centre						
*x* coordinates (mm)	0.8 ± 1.3	1.0	−0.2	1.5	−1.5	3.3
*y* coordinates (mm)	15.3 ± 2.6	14.6	13.0	17.7	11.9	19.0
Patient's left side						
*x* coordinates (mm)	7.0 ± 1.9	7.0	6.0	8.6	3.3	10.0
*y* coordinates (mm)	15.5 ± 3.8	14.1	13.0	19.8	9.5	20.8

The origin of coordinates as shown in Figure 5 is defined by the midline (raphe) and the incisal edges of the central incisors. Negative *x* coordinates are defined as left of midline on the picture, which corresponds to the patient's right side.

### Discussion

The analysis of the proposed treatment options revealed interesting insights in the treatment choices of orthodontists in Switzerland. The case presented in the current study was considered to be a borderline Class II case not only because four premolars and all wisdom teeth were extracted but also because of the good facial aesthetics in combination with a skeletal Class II case. Therefore, either camouflage or a combined orthodontic and orthognathic treatment was a viable treatment option. The majority of orthodontists participating in our study have chosen a comprehensive orthodontic camouflage plan with distalization in the upper arch. Long-term outcomes of Class II adults treated with either camouflage or a combined orthodontic and orthognathic treatment plan have been compared by Mihalik and co-workers [6]. No differences were detected in posttreatment overbite change, but posttreatment overjet enlargement was larger in the surgery group. However, the surgery group included more severe cases. In the same study, the ideal camouflage patient was defined as one with reasonably good aesthetics and with overjet mostly confined to the maxillary dentition and not the skeleton. In the case presented in our study, aesthetics was reasonably good and the profile was not a concern to the patient. All those factors may be sufficient for the majority of orthodontists to choose camouflage treatment over surgery. The expected profile change was expected to be small as the maxillary incisor retroclination required root distalization. Furthermore, extraction spaces of the wisdom teeth provided the necessary space for distalization and correction of the sagittal relationship thus favouring the distalization option which was the treatment of choice by most of the survey participants.

In the literature, the Herbst appliance is also recommended as an effective and predictable device for the correction of Class II malocclusions in adults [2-4,10,11]. Several studies have compared adult Class II cases treated with sagittal split osteotomy and Herbst appliance [3,12], and it was shown that both treatment approaches were successful; however, the surgical approach showed more skeletal effects, whereas the Herbst treatment approach showed more dento-alveolar effects. Although remodelling of the glenoid fossa and the condyle could be detected and skeletal effects could be measured [3,10,12], a recent study [4] showed that only a minimal skeletal effect was left after the retention period. In the current study, only 3.7% have chosen the Herbst treatment approach. Although the Herbst approach is a widely accepted treatment choice, the discrepancy could be attributed to the sample of orthodontists that participated in the study, country-specific treatment strategies and low response rate. A similar study in other countries might therefore show interesting insights in the variability of treatment approaches between countries.

Another common approach in Class II cases, where the lower jaw can be treated without extraction, is the extraction of the upper teeth. Since four premolars and all wisdom teeth had already been extracted, the extraction of any of the remaining upper teeth could not be justified and also was not proposed by any of the practitioners.

The palatal implant was the most often planned skeletal anchorage in about 70% of all distalization concepts. There is evidence that palatal implants are effective and highly reliable with very high success rates and with almost all failures occurring during the healing phase [13-16]. Those advantages make them ideal anchorage devices after successful osseointegration. Nevertheless, the amount of palatal implants planned seems very high in relation to the amount of other TADs used in the current case, especially mini-screws. This might indicate a country-specific trend, which happens to be the country where the palatal implant was developed.

Mini-screws were the second most frequently used appliance as TADs in distalization treatment plans. In contrast to palatal implants, mini-screws are often preferred because they are less expensive, are easier to insert by the orthodontist without the need for the oral surgeon and can be loaded immediately. The survival rates of palatal mini-screws have been shown to be similar with those of the palatal implants [13,17,18].

Within the category, where TADs were used for distalization, most factors were associated with the TAD types proposed. The association among TAD-type, the university and country of specialisation and fixed appliance techniques might suggest that the orthodontists continue to treat their patients as they were educated. However, it has to be kept in mind that multiple comparisons might have introduced false significant association (type 1 errors) and that the coefficient of determination was low (≤0.35), which indicates weak associations (Table 3).

The palatal implant positions showed a large variation in the sagittal plane with very little variation in the transversal plane. Both mid-sagittal [19-21] and paramedian [22-24] positions have been suggested in the literature. In the current survey, the majority of implants were positioned in the mid-sagittal position. Although most palatal implants were inserted in a non-tissue invasive position, the most anteriorly positioned TADs could possibly lead to complications by damaging the roots of the incisors, causing endodontic problems or damaging the incisal nerve depending on the angulation of the implant [25]. However, some less safe TAD positions may be attributed to the fact that some participants did not pay attention to carefully position the TAD and properly adjust the orientation.

Recent digital volumetric imaging studies analysed palatal thickness and revealed that the thickest part of the palate is in the anterior region and that in the posterior region, mini-screws of appropriate lengths can also be placed [24],[26-28], which makes all proposed screw positions reasonable. The thick palatal bone allows wide and long screws to be used, as planned by several participants. Since only palatal screws were used, a certain bias might be introduced by the layout of the webpage because it was initialised with the occlusal view of the upper arch showing the palate.

A clear weakness of the study is the low response rate of 23.5%, which is likely to have introduced non-response bias. An older study in the UK evaluated 77 publications based on mailed questionnaires and found much higher response rates of 64% on average with a range from 17% to 100% [29]. However, over 100 orthodontists participated in our survey, which covers a wide range of orthodontists and treatment philosophies in Switzerland.

One of the reasons for the low response rate might be related to the complexity of a long questionnaire. The literature supports this assumption since shorter questionnaires achieve higher response rates [29]. Also, incentives, such as reminder letters, can increase response rates [29] and were used in our study.

The low response rate could also be assigned to the specific treatment planning challenge which had to be addressed to the clinicians, i.e. retreating an unsuccessfully treated case. For this reason, the figures obtained for the usage of TADs might not represent the exact proportion of the orthodontic community in Switzerland but only reflect a trend of the sample responded. However, due to the complete liberty, a wide spectrum of responses could be acquired, which makes the study more representative. It can also be hypothesised that the variation of treatments identified in the responses and the complete liberty in formulating a treatment plan provided in the questionnaire warrant that the lack of a high response rate was not associated with a specific limitation of questionnaire or possible disagreement with the proposed direction (non-surgical, surgical) of treatment. The latter would have been valid only if the questionnaire limited the choice of responders by forcing a TAD treatment plan and asking for the specific type and location of TAD. Therefore, the results and conclusions of our study should still be externally valid and generalisable to a certain extent for the population of orthodontists in Switzerland.

## Conclusions

The following are the conclusions drawn from the study:

 Camouflage treatment with distalization in the upper jaw using TADs was by far the most popular treatment plan (>75%).

 The most frequent TAD type was the palatal implant (>70%), which was more often placed in the median than the paramedian position with small transversal and wide sagittal range.

 All mini-screw positions were palatal with a median diameter of 2.0 mm and a median length of 10.0 mm and positioned in lateral groups parallel to the alveolar ridge or a median group.

## Competing interests

The authors declare that they have no competing interests.

## Authors’ contributions

TE, CK and GM developed the study protocol, the webpage design and the structured questionnaire together. GM wrote the client and server code of the webpage, administered the webpage and made the data acquisition and interpretation. GM and NP performed the statistical analysis and interpreted the results. GM wrote the manuscript. TE and CK guided and supported GM in every phase of the study. TE, CK and NP revised the manuscript. All authors read and approved the final manuscript and have given the final approval of the version to be published.

## Additional file

## Supplementary Material

Additional file 1 Questions in the case-specific section. Click here for file

## References

[B1] Schatzle M Mannchen R Zwahlen M Lang NP Survival and failure rates of orthodontic temporary anchorage devices: a systematic review Clin Oral Implants Res 2009 15 12 1351 9 10.1111/j.1600-0501.2009.01754.x 19793320

[B2] Ruf S Pancherz H Dentoskeletal effects and facial profile changes in young adults treated with the Herbst appliance Angle Orthod 1999 15 3 239 46 10371429 10.1043/0003-3219(1999)069<0239:DEAFPC>2.3.CO;2

[B3] Ruf S Pancherz H Orthognathic surgery and dentofacial orthopedics in adult Class II Division 1 treatment: mandibular sagittal split osteotomy versus Herbst appliance Am J Orthod Dentofacial Orthop 2004 15 2 140 52 quiz 254–5 10.1016/j.ajodo.2004.02.011 15316468

[B4] Bock NC Ruf S Dentoskeletal changes in adult Class II division 1 Herbst treatment—how much is left after the retention period? Eur J Orthod 2012 15 6 747 53 10.1093/ejo/cjr087 21785003

[B5] Cassidy DW Jr Herbosa EG Rotskoff KS Johnston LE Jr A comparison of surgery and orthodontics in “borderline” adults with Class II, division 1 malocclusions Am J Orthod Dentofacial Orthop 1993 15 5 455 70 10.1016/0889-5406(93)70072-V 8237898

[B6] Mihalik CA Proffit WR Phillips C Long-term follow-up of Class II adults treated with orthodontic camouflage: a comparison with orthognathic surgery outcomes Am J Orthod Dentofacial Orthop 2003 15 3 266 78 10.1067/mod.2003.43 12637899 PMC3556244

[B7] Daskalogiannakis J Glossary of Orthodontic Terms 2000 Quintessence Publishing, Hanover Park, IL

[B8] Buschang PH Carrillo R Ozenbaugh B Rossouw PE 2008 Survey of AAO members on miniscrew usage J Clin Orthod 2008 15 9 513 8 18974458

[B9] Shirck JM Firestone AR Beck FM Vig KW Huja SS Temporary anchorage device utilization: comparison of usage in orthodontic programs and private practice Orthodontics (Chic) 2011 15 3 222 31 22022693

[B10] Ruf S Pancherz H Temporomandibular joint remodeling in adolescents and young adults during Herbst treatment: a prospective longitudinal magnetic resonance imaging and cephalometric radiographic investigation Am J Orthod Dentofacial Orthop 1999 15 6 607 18 10.1016/S0889-5406(99)70285-4 10358242

[B11] Purkayastha SK Rabie AB Wong R Treatment of skeletal class II malocclusion in adults: stepwise vs single-step advancement with the Herbst appliance World J Orthod 2008 15 3 233 43 18834006

[B12] Chaiyongsirisern A Rabie AB Wong RW Stepwise advancement Herbst appliance versus mandibular sagittal split osteotomy: treatment effects and long-term stability of adult Class II patients Angle Orthod 2009 15 6 1084 94 10.2319/110308-556R.1 19852598

[B13] Tsui WK Chua HD Cheung LK Bone anchor systems for orthodontic application: a systematic review Int J Oral Maxillofac Surg 2012 15 11 1427 38 10.1016/j.ijom.2012.05.011 22704592

[B14] Zuger J Pandis N Wallkamm B Grossen J Katsaros C Success rate of paramedian palatal implants in adolescent and adult orthodontic patients: a retrospective cohort study Eur J Orthod 2013 15 1 22 5 doi:10.1093/ejo/cjt003 10.1093/ejo/cjt003 23525601

[B15] Jung BA Kunkel M Gollner P Liechti T Wagner W Wehrbein H Prognostic parameters contributing to palatal implant failures: a long-term survival analysis of 239 patients Clin Oral Implants Res 2012 15 6 746 50 10.1111/j.1600-0501.2011.02197.x 21545530

[B16] Asscherickx K Vannet BV Bottenberg P Wehrbein H Sabzevar MM Clinical observations and success rates of palatal implants Am J Orthod Dentofacial Orthop 2010 15 1 114 22 10.1016/j.ajodo.2008.02.022 20122439

[B17] Karagkiolidou A Ludwig B Pazera P Gkantidis N Pandis N Katsaros C Survival of palatal miniscrews used for orthodontic appliance anchorage: a retrospective cohort study Am J Orthod Dentofacial Orthop 2013 15 6 767 72 10.1016/j.ajodo.2013.01.018 23726326

[B18] Kim YH Yang SM Kim S Lee JY Kim KE Gianelly AA Kyung SH Midpalatal miniscrews for orthodontic anchorage: factors affecting clinical success Am J Orthod Dentofacial Orthop 2010 15 1 66 72 10.1016/j.ajodo.2007.11.036 20122433

[B19] Triaca A Antonini M Wintermantel E Ein neues Titan-Flachschrauben-Implantat zur orthodontischen Verankerung am anterioren Gaumen Inf Orthod Kieferorthop 1992 15 251 7

[B20] Wehrbein H Glatzmaier J Mundwiller U Diedrich P The Orthosystem—a new implant system for orthodontic anchorage in the palate J Orofac Orthop 1996 15 3 142 53 10.1007/BF02191878 8655109

[B21] Stockmann P Schlegel KA Srour S Neukam FW Fenner M Felszeghy E Which region of the median palate is a suitable location of temporary orthodontic anchorage devices? A histomorphometric study on human cadavers aged 15–20 years Clin Oral Implants Res 2009 15 3 306 12 10.1111/j.1600-0501.2008.01647.x 19397643

[B22] Bernhart T Vollgruber A Gahleitner A Dortbudak O Haas R Alternative to the median region of the palate for placement of an orthodontic implant Clin Oral Implants Res 2000 15 6 595 601 10.1034/j.1600-0501.2000.011006595.x 11168253

[B23] Bernhart T Freudenthaler J Dortbudak O Bantleon HP Watzek G Short epithetic implants for orthodontic anchorage in the paramedian region of the palate: a clinical study Clin Oral Implants Res 2001 15 6 624 31 10.1034/j.1600-0501.2001.120611.x 11737107

[B24] AlSamak S Gkantidis N Bitsanis E Christou P Assessment of potential orthodontic mini-implant insertion sites based on anatomical hard tissue parameters: a systematic review Int J Oral Maxillofac Implants 2012 15 4 875 87 22848890

[B25] Fah R Schatzle M Complications and adverse patient reactions associated with the surgical insertion and removal of palatal implants: a retrospective study Clin Oral Implants Res 2013 10.1111/clr.12152 23551587 10.1111/clr.12152

[B26] Gracco A Lombardo L Cozzani M Siciliani G Quantitative cone-beam computed tomography evaluation of palatal bone thickness for orthodontic miniscrew placement Am J Orthod Dentofacial Orthop 2008 15 3 361 9 10.1016/j.ajodo.2007.01.027 18774082

[B27] Gracco A Lombardo L Cozzani M Siciliani G Quantitative evaluation with CBCT of palatal bone thickness in growing patients Prog Orthod 2006 15 2 164 74 17143344

[B28] Alsamak S Psomiadis S Gkantidis N Positional guidelines for orthodontic mini-implant placement in the anterior alveolar region: a systematic review Int J Oral Maxillofac Implants 2013 15 2 470 9 10.11607/jomi.2659 23527349

[B29] Tan RT Burke FJT Response rates to questionnaires mailed to dentists: a review of 77 publications Int Dent J 1997 15 6 349 54 10.1111/j.1875-595X.1997.tb00460.x

