# Supplementary material for: Temporary anchorage device usage: a survey among Swiss orthodontists
Source: Prog Orthod. 2014 Apr 1;15(1):29. doi: 10.1186/s40510-014-0029-x (PMC4047490; doi:10.1186/s40510-014-0029-x)
Supplement: Additional file 1 — Questions in the case-specific section. [file s40510-014-0029-x-S1.docx]

**Questions in the case-specific section:**

1. *“If your treatment plan includes skeletal anchorage devices to solve this specific orthodontic problem, please use the application just below to put the desired screws to the appropriate positions and orientations.*

*Otherwise activate this option: No skeletal anchorage devices needed for this orthodontic problem.”*

- 1. If skeletal anchorage is used an Adobe^®^ Flash^®^ application was shown (Figure 4) that allows to put as many screws and Implants on each of the three intraoral pictures: buccal right and left view in centric occlusion and occlusal view of the upper jaw. Miniscrews, palatal implants, onplants and miniplates and other TADs, which had to be defined later, were available. The position and orientation were registered of each TAD on every intraoral picture was registered.

In case of miniscrew placement:

- - 1. *“Please describe shortly the types, lengths and diameters of the Miniscrews that you have chosen to use:”* as free text.

In case of miniplate placement:

- - 1. *“Please describe shortly the types of miniplates that you have chosen to use:”* as free text.

In case of placement another type of TAD named “other” in the Adobe^®^ Flash^®^ application:

- - 1. *“Please describe shortly the other types of anchorage devices that you have chosen to use:”* as free text.

In case of any TAD placement:

- - 1. *“Please describe shortly the appliances and mechanics you plan to use together with the skeletal anchorage devices:”* as free text.

1. *“Please write VERY SHORTLY how you are going to correct the upper crowding according to your treatment plan?”* as free text.
2. *“Which will be the occlusion on the lateral segments after treatment according to your treatment plan?”* as free text.

**General questions section:**

1. *“Which orthodontic technique are you using routinely?”* choosing among the following options:
   1. *“Straight wire (sliding)”*
   2. *“Level edge wise (loops)”* – Straight wire with loop mechanics
   3. *“Standard edge wise”*
   4. *“Tweed”*
   5. *“Bioprogressive”*
   6. *“Other”*

In case of “other”:

- - 1. *“Other orthodontic technique?”* As free text.

1. *“Are you using self-ligating brackets routinely?”* With *“yes”* or *“no”* as options.
2. *“Which bracket slot sizes or types do you use routinely?”*  choosing among the following options:
   1. *“0.018" ”*
   2. *“0.022" ”*
   3. “other or combination”

In case of “other or combination”:

- - 1. *“Please specify the combinations and/or other slot types that you use:”* As free text.

1. *How many skeletal anchorage devices have you placed approximately in February and March of 2012 per anchorage device type?*
   1. *“Miniscrews:”* as free text or option *“none”*
   2. *“Palatal implants:”* as free text or option *“none”*
   3. *“Miniplates:”* as free text or option *“none”*
   4. *“Palatal onplants:”* as free text or option *“none”*
   5. *“Other skeletal anchorage devices (please list all types with approxiomate numbers):* as free text or option *“none”*
2. *“Country where specialization was obtained:”* as free text.
3. “University where specialization was obtained:” as free text.
4. *“How many years have you been working as orthodontist?”* as free text.
5. *“Which country are you currently working in?”* as free text.
6. *“Do you work in a private practice as:”* choosing among the following options:
   1. *“practice owner”*
   2. *“practice partner”*
   3. *“assistant or associate”*
   4. *“other”*

In case of “other”:

- - 1. *“Otherwise working in private practice?”* as free text.

In case of options 9.1 to 9.4:

- - 1. *“How many years have you been working in private practice?”* As free text.
  1. *“Not working in private practice”*

1. *“Gender:”* With *“Male”* or *“Female”* as options.
2. *“Age:”* As free text.
